# Alcohol consumption and mortality among Canadian drinkers: A national population‐based survival analysis (2000–2017)

**DOI:** 10.1111/dar.13993

**Published:** 2024-12-12

**Authors:** James M. Clay, Russell C. Callaghan, Adam Sherk, Timothy S. Naimi, Tim Stockwell, Mark Asbridge

**Affiliations:** ^1^ Canadian Institute for Substance Use Research University of Victoria Victoria Canada; ^2^ Department of Community Health and Epidemiology Dalhousie University Halifax Canada; ^3^ Department of Psychology University of Victoria Victoria Canada; ^4^ Northern Medical Program University of Northern British Columbia Prince George Canada; ^5^ Canadian Centre on Substance Use and Addiction Ottawa Canada; ^6^ School of Public Health and Social Policy University of Victoria Victoria Canada

**Keywords:** alcohol policy, drinking guidelines, longitudinal study, moderate drinking, mortality

## Abstract

**Introduction:**

Alcohol contributes significantly to global disease burden. Over 50 countries, including Canada, have established low‐risk drinking guidelines to reduce alcohol‐related harm. *Canada's Guidance on Alcohol and Health* (CGAH) was released in 2023. This study examines the relationship between weekly alcohol consumption, CGAH risk zones and mortality patterns among Canadian drinkers aged 15 and older.

**Methods:**

A retrospective cohort study was conducted using data from three cycles of the national, population‐based Canadian Community Health Survey (2000–2006) linked to mortality data up to 2017. The sample included 145,760 respondents aged 15 and older who reported alcohol consumption in the past week. Average weekly alcohol consumption was assessed using the Timeline Followback method (i.e., 7‐day recall). Outcomes included all‐cause mortality, alcohol‐related mortality and mortality from conditions with an alcohol‐attributable fraction ≥15%.

**Results:**

Alcohol consumption was significantly positively associated with increased risks of all‐cause (hazard ratio = 1.01, *p* < 0.001), alcohol‐related (hazard ratio = 1.01, *p* = 0.001) and alcohol‐attributable fraction‐related mortality (hazard ratio = 1.02, *p* < 0.001). Each additional standard drink per week raised mortality risk, with women experiencing a greater increase in risk compared to men.

**Discussion and Conclusion:**

The findings support the CGAH recommendations, highlighting the importance of lower alcohol consumption limits to reduce health risks. Public health efforts should focus on increasing awareness and adherence to these guidelines, particularly among women who face greater mortality risks at higher consumption levels. Ongoing monitoring of alcohol consumption is critical for tracking and evaluating low‐risk drinking guideline effectiveness in reducing alcohol‐related harm.

## 1 INTRODUCTION

The consumption of alcohol results in a wide array of harmful health and social outcomes [1]. It significantly contributes to mortality from various causes, including liver disease, cardiovascular disease and several types of cancer [2, 3]. It is also a leading factor in deaths from accidents, injuries and violence [4]. Globally, alcohol‐related mortality remains a major public health concern [5], with excessive consumption shortening life expectancy and contributing to premature death [6, 7]. Research has consistently demonstrated a dose‐dependent relationship between alcohol consumption and mortality, with higher levels of consumption associated with increased risks of all‐cause mortality and alcohol‐related conditions [8, 9, 10].

While low‐volume drinking has been suggested to confer protection, particularly against cardiovascular disease, recent systematic reviews and meta‐analyses challenge this notion, attributing these findings to methodological flaws [6, 7]. For example *confounding* (i.e., moderate drinkers often have healthier lifestyles, like better diet and exercise, unrelated to alcohol); *selection bias* (i.e., studies often exclude heavy drinkers who died or include those who stopped drinking due to alcohol‐related health problems in the reference group), *misclassification of exposure* (i.e., former drinkers are often incorrectly labelled as lifetime abstainers) and/or *residual bias* (i.e., poor health outcomes of former drinkers are excluded instead of linked to their past drinking) [11].

Canada is no exception to this global trend, with alcohol being one of the most widely used psychoactive substances in the country. For instance, in 2019, nearly 80% of Canadians aged 15 years or older reported *past‐year* drinking [12]. Alcohol is also a leading cause of death and disability in Canada, making a substantial contribution to the nation's substance‐related harms [13]. More specifically, in 2020, alcohol accounted for approximately 40% ($19.7B) of Canada's total substance use costs (including drugs other than alcohol) [13], resulting in a $6.20B annual deficit (alcohol revenues minus harm costs) [14]. This underscores the importance of examining alcohol‐related mortality risk within the Canadian context, where cultural norms, availability and policy approaches may influence drinking behaviours and outcomes.

To help individuals manage their drinking and reduce both immediate and long‐term alcohol‐related harm, over 50 countries have established low‐risk drinking guidelines (LRDG) designed to promote ‘safer’ drinking habits [15]. These guidelines typically provide daily and weekly thresholds and recommend that individuals not exceed them. However, a 2016 review of 37 international LRDGs revealed substantial variation in both standard drink (SD) definitions and risk thresholds across jurisdictions [16]. The study highlighted that the size of an SD ranged from 8 to 20 g of pure ethanol, with 10 g being the most common definition. For ‘low‐risk’ drinking, daily limits varied widely, from 10 to 56 g for men and 10 to 42 g for women. Weekly limits ranged from 98 to 280 g for men and 98 to 140 g for women. These differences reflect diverse cultural, social and public health contexts, but they also pose challenges for cross‐national comparisons. Further, this variability underscores the need for careful consideration of how LRDGs are formulated and communicated across different settings.

The 2011 Canadian LRDGs [17] recommended that women should consume no more than two SDs (each containing 13.45 g of ethanol) per day, with a maximum of 10 per week and no more than three in one sitting. For men, the limits were set at three SDs per day, 15 per week and four in one sitting. This LRDG was based on the relative risk (RR) approach, whereby data from meta‐analyses were used to determine a level of alcohol consumption where the risk all‐cause mortality was no higher than that of a lifetime abstainer (i.e., an RR of ≤1.00) [18].

These guidelines were significantly revised in January 2023 [19]. Specifically, the current Canadian Guidance on Alcohol and Health (CGAH) identifies a continuum of health risks based on alcohol‐caused premature mortality: 0 drinks per week is no risk, 1–2 drinks is low risk, 3–6 drinks is moderate risk and 7+ drinks is increasingly higher risk. It is also recommended not to exceed 2 drinks on any single day. These guidelines apply to everyone as there is no meaningful difference between women and men at up to 7 drinks per week. The CGAH was derived using the lifetime risk approach, whereby the amount of alcohol consumption corresponding to a particular risk threshold was calculated [19, 20]. For example, consuming above 2 SDs per week corresponds to at least a 1 in 1000 risk of premature death, while above 6 SDs per week corresponds to at least a 1 in 100 risk [19].

The CGAH recommendations sparked significant public debate. For instance, an Ipsos poll (*N* = 1350) indicated that most Canadians felt the new guidance ‘*lacked credibility’* and employed ‘*fear‐mongering tactics’* [21]. Given that public misunderstanding of research can undermine the impact of scientific guidance and considering the importance of LRDGs in national alcohol‐control strategies, it is crucial to evaluate LRDGs using real‐world data. Surprisingly, despite the widespread use of LRDGs by many jurisdictions, scant empirical studies have assessed the relations between LRDG‐defined alcohol consumption and subsequent population‐level alcohol‐related harms [22, 23, 24].

This paper aims to address this gap by investigating the link between alcohol consumption, aligned with CGAH risk zones, and mortality among Canadian drinkers aged 15 and older. Specifically, using data from a large Canadian population health survey linked to vital statistics, we conducted a longitudinal analysis of a cohort of drinkers. Our approach enabled the creation of accurate, population‐representative estimates of mortality risk, based on individual‐level measures of alcohol use, with up to 17 years of follow‐up for alcohol‐related mortality. To ensure accuracy, we restricted the analysis to current drinkers, minimising selection biases commonly introduced by including lifetime abstainers or former drinkers as a reference group [11]. Additionally, we accounted for several known confounders using statistical adjustments.

## 2 METHODS

### 2.1 Data source

We pooled three cycles of the Canadian Community Health Survey (CCHS): 1.1 (2000/01), 2.1 (2003/04) and 3.1 (2005/06), that is, CCHS 2000–2006. The CCHS is a national cross‐sectional survey conducted by Statistics Canada. It gathers information on sociodemographic characteristics, health status and healthcare utilisation. The survey targets Canadians aged 12 and older living in private dwellings, covering over 96% of the Canadian population, excluding individuals who are full‐time members of the Canadian Forces, those residing in institutions, and people living on Indigenous reserves and in certain remote areas [25]. The response rates for these surveys ranged from approximately 79–85% [26, 27, 28].

Statistics Canada has linked the CCHS to administrative hospitalisation and mortality data [25]. Approximately 85% of CCHS respondents consented to have their data linked to administrative datasets. Of these, 96% were probabilistically linked to the Derived Record Depository within the Social Data Linkage Environment at Statistics Canada. This linkage was conducted using given and family names, birthdates, sex and postal codes. Health administrative datasets were deterministically linked to this environment using unique identifiers (e.g., health card number), achieving a high linkage rate of 99.8% for deaths (Canadian Vital Statistics Death Database 2000–2017) [29]. A study analysing CCHS data linked to mortality records reported very low error rates, with false positives at 0.04% and false negatives at 2.43% [30].

### 2.2 Ethics

This study used secondary data from Statistics Canada. Thus, no additional ethical approval was required. All procedures comply with Statistics Canada's data use guidelines and adhere to the principles of the World Medical Association Declaration of Helsinki.

### 2.3 Study sample

We pooled 400,055 respondents from the CCHS 2000–2006. We excluded respondents who did not agree to be linked to administrative health records (*n* = 50,985). We also excluded those who were under 15 years of age and respondents of all ages who did not report drinking in the past week (*n* = 203,310). This left 145,760 cases for analysis from CCHS 1.1 (*n* = 48,495), CCHS 2.1 (*n* = 47,355) and CCHS 3.1 (*n* = 49,910). The study sample included 78,180 men and 67,585 women.^1^

We restricted our sample to drinkers as previous research has demonstrated that using lifetime abstainers or former drinkers as a reference group can introduce selection biases, resulting in attenuated or misleading relationships between alcohol and health [11]. Many individuals categorised as ‘abstainers’ report prior alcohol consumption at earlier time points, a misclassification known as ‘former drinker bias’ [31, 32]. Additionally, non‐drinkers often have healthier lifestyle factors unrelated to alcohol [33]. Furthermore, some abstainers are ‘sick quitters’, individuals who stop drinking due to declining health, particularly in older age, which can reverse the apparent direction of causation and obscure true relationships [34].

### 2.4 Exposures

Self‐report alcohol use was measured in the CCHS using a seven‐day Timeline Follow back (TLFB‐7) approach, which has been shown to be more accurate and reliable compared to measures that assess alcohol use over longer time periods [35, 36, 37]. Participants were given instructions to convert various types of alcoholic beverages into Canadian SDs and then asked to recall their alcohol consumption over the past week, indicating the number of SDs consumed each day, starting from the previous day and moving backward through the week. Thus, the volume of alcohol consumption was defined as the number of Canadian SDs consumed in the week leading up to the interview. This data was then classified into four categories [19]: low volume (1–2 drinks per week), medium volume (3–6 drinks per week), high volume (7–15 drinks per week) and excess volume (>15 drinks per week). These corresponded to risk levels in CGAH, except that the ‘increasingly higher risk’ category (7+ drinks per week) was divided into two categories.

Self‐report alcohol use is routinely under‐reported in cohort studies [38, 39, 40]. For example, self‐report alcohol use data from the 2019 Canadian Alcohol and Drugs Survey accounted for just 38% of recorded alcohol sales [40]. Therefore, we used Statistics Canada's population data [41] and data on litres of pure ethanol consumed per capita [42], to calculate the average per capita alcohol consumption, expressed as SDs per week, across the CCHS interview period (2000–2006). An overall correction factor of 1.21 (8.35 SDs/6.92 SDs) was applied to adjust the self‐report data up to match the sales data.^2^ Models were estimated using both self‐report and sales‐adjusted data.

### 2.5 Outcomes

We examined all‐cause and alcohol‐related mortality using CCHS data linked to Canadian Vital Statistics Death Database 2010–2017 data. All‐cause mortality includes death by any cause. Alcohol‐related mortality was defined in two ways: (i) any acute or chronic condition that is causally associated with alcohol as identified by the Centers for Disease Control and Prevention Alcohol‐Related Disease Impact project [43]; and (ii) any acute or chronic conditions which are causally associated with alcohol with an alcohol‐attributable fraction (AAF) ≥ 15%. This approach captures a broad range of conditions with any level of alcohol involvement and a narrower subset of conditions with a stronger causal relationship to alcohol, ensuring a comprehensive, yet focused analysis of alcohol‐related mortality.

The AAF methodology has been described in full elsewhere [44]. Briefly, AAFs represent an estimate of the proportion of each health condition that can be attributed to alcohol consumption (i.e., the proportion of incidents that would not have occurred if alcohol had not been consumed). Therefore, AAF ≥15% indicates that at least 15% of a particular disease, injury or condition in the population is attributable to alcohol consumption. The calculation of AAFs was automated using InterMAHP [44, 45], which incorporated data on per capita consumption, drinking prevalence, binge drinking prevalence, abstention rates and RR functions that detail the relationship between alcohol consumption and the risk of various alcohol‐related conditions. Calculated AAFs were for Canada in 2017 and were from the Canadian Substance Use Costs and Harms study [13].

To provide additional context, we also estimated the AAF for total mortality by dividing the number of alcohol‐attributable deaths in Canada in 2020 (*n* = 17,098) [13] by the total number of deaths in the same year (*n* = 307,205) [46]. Based on this calculation, approximately 5.57% of all deaths in Canada in 2020 were attributable to alcohol consumption.

International Classification of Diseases 10th Revision codes are included in Appendix A. Time at risk was operationalised as the time from the CCHS survey date until the date of death or the end of the follow‐up period.

### 2.6 Covariates

Potential confounders included factors that could plausibly be related to our outcome: biological sex (men/women), age (continuous), race (White, East Asian, South Asian, Black, South East Asian, West Asian, Aboriginal and other), province, rurality (a dichotomous Statistics Canada‐derived variable where urban is defined as population concentration ≥ 1000 and a population density ≥ 400 per square km based on census population counts), survey cycle (categorical), self‐perceived health (poor, fair, good, very good, and excellent), education (less than secondary, secondary, some post‐secondary and post‐secondary), income adequacy (household income and size were used to designate the following categories: lowest income; lower middle income; middle income; upper middle income; highest income) and smoking status (never, former, current).

### 2.7 Statistical analysis

Descriptive statistics (means, standard deviations and proportions) were calculated for our variables of interest and select demographic variables. Cox proportional hazard models were used to estimate hazard ratios (HR) and 95% confidence intervals (CI) for the association between alcohol consumption and death. We used a continuous measure of alcohol use in our models. We first regressed our outcomes on alcohol use, controlling for CCHS cycle, province, rurality, age, sex and race (model 1). We then added self‐perceived health to our models (model 2), before adding education and income adequacy (model 3), then smoking status (model 4). For brevity, results for models 1, 2 and 3 are reported in Appendix B. Given that most respondents (90.70%) were White and that some racial groups made up less than 1% of the sample, a dichotomous White/non‐White variable was used in our analyses. We also tested for non‐linear associations by including squared and cubed in the models but these tests did not reveal any significant deviations from a linear relationship.

Analyses were stratified by sex to account for known biological and behavioural differences in alcohol metabolism, consumption patterns and associated health risks between men and women [47, 48, 49]. This allowed for sex‐specific patterns in the relationship between alcohol consumption and mortality to be examined. To enhance the interpretability of our results while preserving the precision of a continuous model, we calculated weighted average HRs and 95% CIs for our three outcomes across four predefined CGAH weekly drinking groups: low (1–2 SDs), medium (3–6 SDs), high (7–15 SDs), excess (>15 SDs). These averages were derived from the continuous models by weighting HRs and 95% CIs at each 1 SD interval (up to 55+ SDs) by the number of respondents in each interval. This approach preserves the granularity and accuracy of a continuous analysis while providing interpretable group‐level results, avoiding the information loss and biases that arise from categorising continuous data [50]. In this framework, an HR from a continuous model reflects the change in mortality risk per unit increase in alcohol consumption, and the weighted averages offer meaningful comparisons across the CGAH‐defined drinking groups without relying on a fixed reference group.

Statistical analyses were conducted using Stata (version 18) and figures were generated using *ggplot2* (version 3.5.0) for R (version 4.3.3). We applied bootstrapping with 500 repetitions, following the procedures outlined by Statistics Canada, to account for the complex design of the CCHS [29]. Statistics Canada linkage survey weights, scaled by a third to combine three CCHS cycles [51], were used to adjust for nonresponse, respondents who were not linked or did not consent to share and link their survey responses, and to generate population‐representative estimates. Finally, as neither the study nor analysis plan was pre‐registered on a publicly available platform, the results should be considered exploratory.

## 3 RESULTS

Our study included 18,160 incidents of all‐cause mortality, 7210 incidents of alcohol‐related mortality and 1315 incidents of mortality due to a condition with an AAF ≥15% among 145,760 respondents (*M*_age_ = 43.30, SD_age_ = 15.57). One‐third of the sample fell into the low‐ (32%) and medium‐volume (31%) groups, while one‐quarter were in the high‐volume group (27%) and one‐tenth in the excess‐volume group (10%). Table 1 presents the sociodemographic characteristics of the drinking population (15 years and older) and descriptive statistics (means, standard deviations and proportions) for the main study variables. Table 2 summarises the results from the continuous models, presenting HRs, 95% CIs, standard errors (SE) and *p*‐values for the associations between alcohol consumption and mortality.

**TABLE 1:** Sociodemographic characteristics of the sample and descriptive statistics for study variables.

Variable	Combined (SD)	Men (SD)	Women (SD)
*N*	145,760	78,180	67,585
Self‐report drinks per week	6.92 (7.89)	8.52 (9.00)	4.83 (5.11)
Sales‐adjusted drinks per week^a^	13.66 (15.57)	16.80 (17.76)	9.54 (10.09)
5 + drinks at least monthly	56.68%	66.56%	43.74%
Province			
NFL	1.68%	1.68%	1.47%
PEI	0.34%	0.35%	0.34%
NS	2.66%	2.66%	2.62%
NB	2.04%	2.04%	1.88%
QB	25.48%	25.47%	27.07%
ON	38.90%	38.90%	37.83%
MN	3.19%	3.19%	3.16%
SK	2.78%	2.78%	2.73%
AB	9.76%	9.75%	9.05%
BC	12.94%	12.95%	13.61%
YU	0.10%	0.10%	0.09%
NT	0.11%	0.12%	0.09%
NU	0.03%	0.03%	0.04%
Urban (vs. rural)	82.00%	81.39%	82.82%
Age (SD)	43.30 (15.97)	43.13 (15.43)	43.50 (16.66)
Race			
White	90.70%	89.66%	92.06%
East Asian	1.89%	2.09%	1.62%
South Asian	1.36%	1.83%	0.75%
Black	1.01%	0.99%	1.05%
South East Asian	0.92%	1.04%	0.77%
West Asian	0.43%	0.53%	0.30%
Aboriginal	1.15%	1.08%	1.24%
Other	2.23%	2.47%	1.90%
Missing	0.30%	0.32%	0.30%
Education			
Less than secondary	12.53%	13.31%	11.54%
Secondary school	15.28%	14.82%	15.89%
Some post‐secondary	7.62%	7.31%	8.04%
Post‐secondary	61.42%	60.90%	62.09%
Missing	3.14%	3.68%	2.45%
Income adequacy			
Lowest (1)	1.64%	1.55%	1.75%
Lower middle (2)	3.25%	2.60%	4.10%
Middle (3)	13.38%	12.67%	14.30%
Upper middle (4)	30.82%	30.88%	30.75%
Highest (5)	34.89%	36.18%	33.20%
Missing	16.01%	16.11%	15.89%
Marital status			
Married	53.44%	55.21%	51.12%
Common‐law	11.13%	10.85%	11.52%
Widowed	3.21%	1.57%	5.35%
Separated	2.36%	2.11%	2.67%
Divorced	4.62%	3.70%	5.82%
Single	25.21%	26.52%	23.51%
Missing	0.04%	0.03%	0.06%
Self‐perceived health			
Excellent	25.55%	25.27%	25.92%
Very good	39.85%	39.05%	40.88%
Good	26.87%	27.63%	25.88%
Fair	6.31%	6.52%	6.02%
Poor	1.40%	1.47%	1.32%
Missing	0.02%	0.04%	0.00%
Smoking status			
Never	24.25%	21.43%	27.52%
Former	46.42%	48.29%	44.26%
Current	29.25%	30.18%	28.16%
Missing	0.08%	0.10%	0.06%

*Note*: Drinks per week = Canadian standard drinks per week (1 standard drink = 13.45 g of ethanol).

Abbreviations: AB, Alberta; BC, British Columbia; MN, Manitoba; NB, New Brunswick; NFL, Newfoundland and Labrador; NS, Nova Scotia; NT, Northwest Territories; NU, Nunavut; ON, Ontario; PEI, Prince Edward Island; QB, Quebec, SK, Saskatchewan; YU, Yukon.

^a^ Data were adjusted for underreporting of alcohol use using alcohol sales data.

**TABLE 2:** Summary of the Cox proportional hazard models estimating the association between alcohol consumption and mortality, adjusting for CCHS cycle, province, rurality, age, sex, race, education, income adequacy and smoking status.

	Men					Women					Combined				
				95% CI					95% CI					95% CI	
Outcome	HR	SE	*p*	LL	UL	HR	SE	*p*	LL	UL	HR	SE	*p*	LL	UL
Self‐report															
All‐cause	1.008	0.001	<0.001	1.005	1.010	1.020	0.004	<0.001	1.011	1.029	1.009	0.001	<0.001	1.007	1.012
Alcohol‐related	1.005	0.002	0.010	1.001	1.009	1.013	0.006	0.016	1.002	1.025	1.006	0.002	0.001	1.003	1.010
AAF ≥15%^b^	1.016	0.003	<0.001	1.011	1.021	1.041	0.009	<0.001	1.024	1.058	1.017	0.003	<0.001	1.012	1.022
Sales‐adjusted^a^															
All‐cause	1.004	0.001	<0.001	1.003	1.005	1.010	0.002	<0.001	1.006	1.015	1.005	0.001	<0.001	1.003	1.006
Alcohol‐related	1.003	0.001	0.010	1.001	1.005	1.007	0.003	0.016	1.001	1.012	1.003	0.001	0.001	1.001	1.005
AAF ≥15%^b^	1.008	0.001	<0.001	1.006	1.011	1.021	0.004	<0.001	1.012	1.029	1.009	0.001	<0.001	1.006	1.011

*Note*. AAF, alcohol‐attributable fraction (which denotes the proportion of a health outcome which is caused by alcohol); CI, confidence interval; HR, hazard ratio; LL and UL indicate the lower and upper limits of a confidence interval, respectively. In continuous models, the HR reflects the change in mortality risk per unit increase in consumption.

^a^ Data were adjusted for underreporting of alcohol use using alcohol sales data.

^b^ AAFs represent an estimate of the proportion of disease or injurythat can be attributed to alcohol consumption (i.e., the proportion ofincidents that would not have occurred if alcohol had not been consumed). Therefore, AAF ≥ 15% indicates that at least 15% of a particular disease, injury, or condition in the population is attributable to alcohol consumption.

### 3.1 All‐cause mortality

**FIGURE 1:**
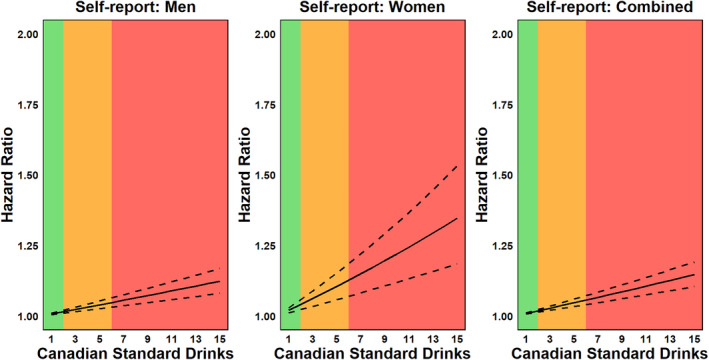
Hazard ratio functions for the relationship between self‐report alcohol consumption (Canadian standard drinks per week) and the risk of all‐cause morality across the continuum of risk associated with weekly alcohol use as per Canada's 2023 Guidance on Alcohol and Health. 1 Canadian standard drink = 13.45 g of ethanol. The solid line represents the hazard ratio, while dashed lines represent 95% confidence intervals. The shaded areas correspond to the risk levels defined by Canada's Guidance on Alcohol and Health: green = low risk (1–2 drinks per week), amber = moderate risk (3–6 drinks per week) and red = high risk (7+ drinks per week). For detailed point estimates, including standard errors and *p*‐values, see Table 2.

Figure 1 and Table 2 illustrate the significant positive relationship between self‐reported alcohol use in the 7 days prior to the CCHS interview and the risk of all‐cause mortality. Among men aged 15 years and older who reported drinking, each 1 SD increase in weekly alcohol consumption was associated with a 1% increase in mortality risk (HR = 1.008, 95% CI 1.005–1.010, *p* < 0.001). For women aged 15 and older, the same increase in alcohol consumption corresponded to a 2% increase in mortality risk (HR = 1.020, 95% CI 1.011–1.029, *p* < 0.001). In the combined sample, each 1 SD increase in weekly alcohol consumption was associated with a 1% increase in the risk of death (HR = 1.009, 95% CI 1.007–1.012, *p* < 0.001).

### 3.2 Alcohol‐related mortality

**FIGURE 2:**
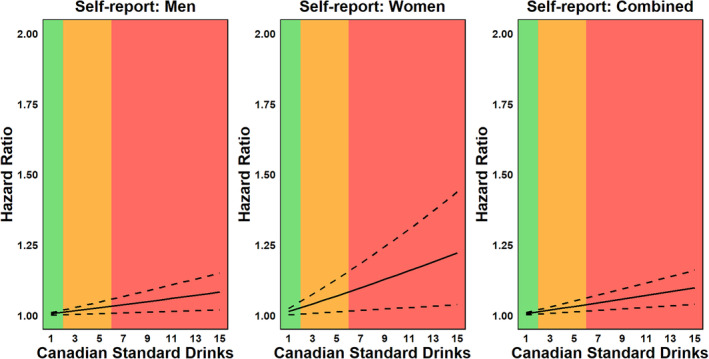
Hazard ratio functions for the relationship between self‐report alcohol consumption (Canadian standard drinks per week) and alcohol‐related mortality across the continuum of risk associated with weekly alcohol use as per Canada's 2023 guidance on alcohol and health. 1 Canadian standard drink = 13.45 g of ethanol. The solid line represents the hazard ratio, while dashed lines represent 95% confidence intervals. The shaded areas correspond to the risk levels defined by Canada's Guidance on Alcohol and Health: green = low risk (1–2 drinks per week), amber = moderate risk (3–6 drinks per week) and red = high risk (7+ drinks per week). For detailed point estimates, including standard errors and *p*‐values, see Table 2.

Figure 2 and Table 2 show the significant positive association between self‐reported alcohol consumption and risk of death due to alcohol‐related mortality. For men, each 1 SD increase in weekly alcohol consumption was associated with a 1% increase in mortality risk (HR = 1.005, 95% CI 1.001–1.009, *p* = 0.002). Among women, the same increase in alcohol consumption resulted in a 1% rise in mortality risk (HR = 1.013, 95% CI 1.002–1.025, *p* = 0.016). When considering the combined sample, each 1 SD increase in weekly alcohol consumption corresponded to a 1% increase in the risk of death (HR = 1.006, 95% CI 1.003–1.010, *p* = 0.001).

### 3.3 Mortality due to a condition with an AAF ≥15%

**FIGURE 3:**
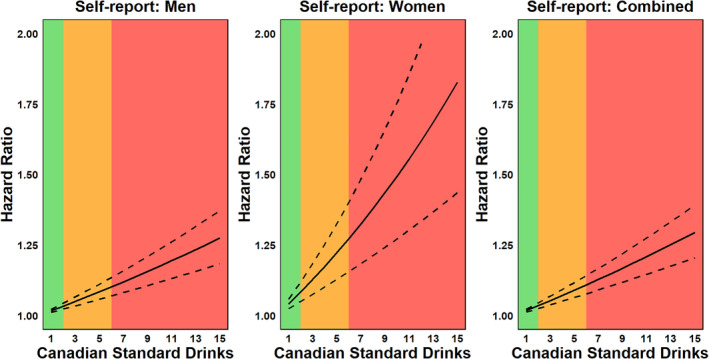
Hazard ratio functions for the relationship between self‐report alcohol consumption (Canadian standard drink per week) and mortality due to a health condition with an alcohol‐attributable fraction (AAF) ≥ 15% across the continuum of risk associated with weekly alcohol use as per Canada's 2023 Guidance on Alcohol and Health. 1 Canadian standard drink = 13.45 g of ethanol. The solid line represents the hazard ratio, while dashed lines represent 95% confidence intervals. The shaded areas correspond to the risk levels defined by Canada's Guidance on Alcohol and Health: green = low risk (1–2 drinks per week), amber = moderate risk (3–6 drinks per week) and red = high risk (7+ drinks per week). AAFs represent an estimate of the proportion of disease or injury that can be attributed to alcohol consumption (i.e., the proportion of incidents that would not have occurred if alcohol had not been consumed). Therefore, AAF ≥15% indicates that at least 15% of a particular disease, injury or condition in the population is attributable to alcohol consumption. For detailed point estimates, including standard errors and *p*‐values, see Table 2.

Figure 3 and Table 2 illustrate the significant positive relationship between self‐reported drinking and mortality due to a condition with an AAF ≥15%. For men, each 1 SD increase in weekly alcohol consumption corresponded to a 2% increase in mortality risk (HR = 1.016, 95% CI 1.011–1.021, *p* < 0.001). For women in the same age group, this increase was 4% (HR = 1.041, 95% CI 1.024–1.058, *p* < 0.001). When considering both men and women together, each 1 SD increase in weekly alcohol consumption was associated with a 2% rise in the risk of death (HR = 1.017, 95% CI 1.012–1.022, *p* < 0.001).

### 3.4 Weighted average mortality risk across drinking groups

Table 3 presents the weighted average risk of mortality by drinking group across each outcome for both self‐report and sales‐adjusted data. These weighted averages were calculated based on HRs and 95% CIs from the continuous models, adjusted by the number of respondents in each drinking group. Overall, the data indicate a dose–response relationship between alcohol consumption and increased risk of mortality across different outcomes. Higher levels of alcohol consumption are consistently associated with increased HRs for all‐cause mortality, alcohol‐related mortality and conditions with a high AAF (≥ 15%).

**TABLE 3:** Weighted average relationship between alcohol consumption and mortality across the continuum of risk associated with weekly alcohol use as per Canada's 2023 guidance on alcohol and health, adjusting for CCHS cycle, province, rurality, age, sex, race, education, income adequacy and smoking status.

	Low: 1–2 SDs			Medium: 3–6 SDs			High: 7–15 SDs			Excess: 15+ SDs		
Outcome	HR	95% CI		HR	95% CI		HR	95% CI		HR	95% CI	
		LL	UL		LL	UL		LL	UL		LL	UL
Self‐report												
All‐cause	1.013	1.010	1.017	1.040	1.029	1.051	1.093	1.067	1.121	1.270	1.188	1.359
Alcohol‐related	1.009	1.004	1.014	1.027	1.011	1.043	1.063	1.025	1.102	1.176	1.068	1.297
AAF ≥15%^a^	1.025	1.018	1.032	1.077	1.054	1.099	1.184	1.129	1.242	1.582	1.386	1.811
Sales‐adjusted^b^												
All‐cause	1.007	1.005	1.009	1.020	1.014	1.026	1.046	1.033	1.059	1.128	1.091	1.166
Alcohol‐related	1.005	1.002	1.007	1.014	1.006	1.022	1.031	1.013	1.050	1.085	1.034	1.139
AAF ≥15%^a^	1.013	1.009	1.016	1.038	1.027	1.049	1.089	1.063	1.115	1.256	1.177	1.341

*Note*. SD = Canadian standard drink (1 SD = 13.45 g of ethanol). AAF, alcohol‐attributable fraction (which denotes the proportion of a health outcome which is caused by alcohol); CI, confidence interval; HR, hazard ratio; LL and UL indicate the lower and upper limits of a confidence interval, respectively. In continuous models, the HR reflects the change in mortality risk per unit increase in consumption, with weighted average HRs comparing different drinking levels rather than a fixed reference group.

^a^ AAFs represent an estimate of the proportion of disease or injury that can be attributed to alcohol consumption (i.e., the proportion of incidents that would not have occurred if alcohol had not been consumed). Therefore, AAF ≥15% indicates that at least 15% of a particular disease, injury, or condition in the population is attributable to alcohol consumption.

^b^ Data were adjusted for underreporting of alcohol use using alcohol sales data.

### 3.5 Sensitivity analysis

We conducted a sensitivity analysis by adding covariates to our models and comparing HRs across different model specifications to test the robustness of our findings. The covariates included in the analysis were: CCHS cycle, province, rurality, age, sex and race (model 1); self‐perceived health (model 2); education and income adequacy (model 3) and smoking status (model 4). As shown in Appendix B, the observed relationships between alcohol consumption and mortality outcomes are robust as they were not significantly influenced by the covariates considered in this study. However, smoking status tended to attenuate the observed relationships, highlighting its role as an important confounder in this context.

## 4 DISCUSSION

This study examined the association between alcohol consumption and mortality among Canadian drinkers aged 15 years and older, using data from a large Canadian population survey (i.e., the CCHS) linked to administrative death records. These data provide evidence of a significant positive and dose‐dependent, relationship between alcohol consumption and increased risk of all‐cause mortality, alcohol‐related mortality and mortality due to conditions with a high AAF (≥ 15%). To the best of our knowledge, this is one of the first empirical studies to investigate CGAH adherence and subsequent population‐level alcohol‐related harm. Importantly, this study provides evidence supporting the updated CGAH, specifically validating its continuum of risk thresholds and providing empirical evidence to support its recommendations.

The results from our study generally support the updated CGAH, which cautioned that all levels of alcohol consumption carry some risk while recommending that people in Canada consider reducing their drinking. The dose–response relationship observed in this study—where higher levels of alcohol consumption consistently correspond to increased mortality risk—aligns with the continuum of risk approach put forward by the CGAH. For example, the relationship between self‐reported alcohol use and mortality due to a condition with an AAF ≥15% showed clear dose‐dependent effects, with the risk of death increasing from 3% in the low‐risk group, to 8% (medium‐risk), 18% (high‐risk) and 58% (excess‐risk). These findings support the CGAH recommendations to limit alcohol consumption to reduce health risks. For instance, our data show a marked reduction in mortality risk when individuals follow the updated guidelines, which advise a maximum of 6 SDs per week for moderate risk, compared to (for example) the 2011 recommendation for men, which allowed a maximum of 15 SDs per week.

This evidence underscores the importance of public health messaging which aims to empower the public to reduce their alcohol use to mitigate health risks associated with drinking. Public education campaigns and policy interventions should aim to increase knowledge and awareness of these guidelines to mitigate or reduce the health risks associated with alcohol consumption. Our findings directly contribute to the CGAH framework by providing population‐level evidence that highlights the health benefits of adhering to its recommendations.

Our findings also align with previous research by showing a consistent gradient in the relationship between alcohol and mortality. For instance, meta‐analyses [6, 7] and Mendelian randomisation studies [52, 53] have consistently reported increased mortality risks with higher alcohol consumption, supporting the view that no level of alcohol use is entirely safe [54]. These studies also highlight differential risks for men and women, with women experiencing higher RRs at equivalent consumption levels. Similarly, our study also found that women experience a more pronounced increase in the risk of death compared to men as alcohol consumption rises. Whereas, the pattern for men closely mirrored the overall results, likely because the majority of alcohol‐related deaths occur among men. This is consistent with previous evidence showing that women experience more negative effects of alcohol consumption for a given volume of alcohol intake, including risks of physical illness, cognitive impairments and negative social consequences [55, 56]. Erol and Karpyak [47] provide a comprehensive review of potential reasons for this disparity, noting (e.g.) that women achieve higher blood alcohol concentrations after consuming the same amounts of alcohol as men. Given this evidence, and the shift away from gender‐specific guidance as seen in the CGAH, it is crucial to prioritise public health messages that inform women about the heightened risk associated with alcohol consumption.

The association between alcohol consumption and alcohol‐related mortality was not stronger than that for all‐cause mortality, likely due to the inclusion of conditions with lower AAFs in the alcohol‐related mortality category, diluting the observed effect. However, the overlapping 95% CIs for all‐cause and alcohol‐related mortality suggest that the differences in point estimates between these outcomes may not be statistically significant. Therefore, this finding highlights the pervasive impact of alcohol on mortality, regardless of whether the cause of death is directly or indirectly alcohol‐attributable.

Our study had several strengths. First, the use of a large, nationally representative sample with extensive follow‐up data allows for robust population‐level risk estimation. For instance, linking population‐based survey data to administrative health records ensured high‐quality outcome measures. Second, we improved the accuracy of consumption estimates by aligning the self‐reported data with sales data. This adjustment was used to reduce measurement error and tended to attenuate the relationship between alcohol use and mortality risk. Third, excluding lifetime abstainers and former drinkers minimised selection bias, which can distort associations between alcohol consumption and health outcomes. Fourth, we also statistically controlled for known confounders to ensure that the observed associations accurately reflected the relationship between alcohol use and mortality. Fifth, stratification by sex accounted for biological and behavioural differences in alcohol metabolism and associated health risks between men and women. Finally, we applied complex survey weights to adjust for nonresponse and non‐linkage.

We also acknowledge several limitations. First, self‐reported consumption data, even after adjustment, likely underestimates true alcohol intake. For example, heavy drinking is linked to non‐response bias [57] and the short recall period may have excluded infrequent drinkers. Furthermore, the blanket sales adjustment may not account for group‐specific underreporting, such as among younger drinkers or those with impression management bias [58, 59]. We lacked the data to estimate individual‐level under‐reporting; future research should address this in the CCHS. Second, our sample was limited to past‐week drinkers aged 15 years and older, which excluded >50% of the original sample. This exclusion was necessary to minimise selection bias [11] and to focus on individuals within the CGAH risk zones. Excluding abstainers makes our estimates conservative, as comparisons with an unbiased abstainer group (if available) would likely yield higher HRs. However, excluding infrequent drinkers (i.e., past‐year drinkers who did not report drinking in the past week) further reduced the sample size and limited the generalisability of our findings to the broader population of drinkers. Nonetheless, while the CCHS provides past‐year drinking frequency data, it lacks quantity measures, making it incompatible with our quantity‐frequency approach. Additionally, such a group would likely be heterogeneous (e.g., including both infrequent and episodic heavy drinkers), further reducing data interpretability. Future studies could explore incorporating non‐drinkers in a way to addresses selection biases, and the CCHS should consider adding quantity‐based measures for infrequent drinkers. These limitations should be considered when interpreting the results. Third, we did not account for specific drinking patterns, such as binge drinking, which poses distinct health risks [60]. Fourth, some analyses focused on all‐cause mortality, including deaths from conditions that are not causally linked to alcohol consumption. This may also underestimate the association between alcohol use and mortality per our other analyses. Finally, the observational nature of this study precludes causal inference.

## 5 CONCLUSION

This study provides compelling evidence that increasingly higher levels of alcohol consumption among drinkers are associated with increased risks of all‐cause mortality, alcohol‐related mortality and mortality due to conditions with a high AAF, for both men and women. These findings align with, and provide empirical validation for, the updated CGAH recommendations, demonstrating that even low levels of alcohol consumption carry some risk and highlighting the substantial reductions in mortality risk achieved by adhering to CGAH's lower consumption thresholds. These results underscore the need for public health policies that promote adherence to evidence‐based drinking guidelines to mitigate alcohol‐related health problems [61]. Furthermore, they emphasise the importance of targeted public health messaging to increase awareness of the CGAH risk zones and empower individuals to make informed decisions about their alcohol consumption.

These findings also highlight the importance of real‐world data in refining and evaluating drinking guidelines. By providing population‐level evidence, this study contributes to the ongoing validation of the CGAH and supports its use as a framework for reducing alcohol‐related harm. Continued surveillance of alcohol consumption is crucial for monitoring its health and social impacts and assessing the effectiveness of strategies aimed at reducing alcohol‐attributable mortality. Regular evaluation of LRDGs using empirical data will help ensure their relevance and effectiveness in promoting public health.

## AUTHOR CONTRIBUTION

Conceptualization: RCC, MA; Data curation: JMC, AS; Formal Analysis: JMC, AS; Funding acquisition: RCC, MA; Methodology: JMC, RCC, AS, TSN, TS, MA; Project administration: JMC, RCC, MA;Supervision: RCC, TSN, TS, MA; Validation: JMC; Visualization: JMC; Writing – original draft: JMC; Writing – review & editing: JMC, RCC, AS, TSN, TS, MA. Each author certifies that their contribution to this work meets the standards of the International Committee of Medical Journal Editors.

## FUNDING INFORMATION

This study was funded by the Canadian Institutes for Health Research Catalyst Grant (FRN: 180925).

## CONFLICT OF INTEREST STATEMENT

The authors declare no conflicts of interest.

## Supporting information


**Data S1:** Supporting Information

## Data Availability

The data supporting the findings of this study are accessible through Statistics Canada. Access to these data is restricted and subject to licensing agreements. Data can be obtained from Statistics Canada's Research Data Centres (https://www.statcan.gc.ca/en/microdata/data-centres) with appropriate permissions from Statistics Canada.

## Endnotes

^1^
Due to the random rounding of the counts to base 5, as per Statistics Canada Protocols, they may not add up to the overall sample in our study.

^2^
The sales‐adjusted data does not exactly match the actual sales data as a uniform correction factor was applied to all individuals. This assumes that all individuals under‐report their consumption by the same amount, which may not be the case. We lacked the data needed to estimate individual‐level under‐reporting.
