# Supplementary material for: Alcohol consumption and mortality among Canadian drinkers: A national population‐based survival analysis (2000–2017)
Source: Drug Alcohol Rev. 2024 Dec 12;44(2):434–47. doi: 10.1111/dar.13993 (PMC11814365; doi:10.1111/dar.13993)
Supplement: Supplementary file 1 — Data S1: Supporting Information [file DAR-44-434-s001.docx]

# Supporting Information

Supplement to:

Alcohol consumption and mortality among Canadian drinkers: A national population-based survival analysis (2000–2017)

**Appendix A1.** International Classification of Diseases (ICD-10) codes for chronic and acute conditions which are wholly and partially attributable to alcohol as identified by the Centers for Disease Control and Prevention (CDC, 2021).

| **Cause of death** | **ICD-10 code** |
| --- | --- |
| Alcoholic psychosis | F10.3-F10.9 |
| Alcohol abuse | F10.0, F10.1 |
| Alcohol dependency syndrome | F10.2 |
| Alcohol polyneuropathy | G62.1 |
| Degradation of nervous system due to alcohol | G31.2 |
| Alcoholic myopathy | G72.1 |
| Alcohol cardiomyopathy | I42.6 |
| Alcoholic gastritis | K29.2 |
| Alcoholic liver disease | K70.0-K70.4, K70.9 |
| Alcohol-induced acute pancreatitis | K85.2 |
| Alcohol-induced chronic pancreatitis | K86.0 |
| Fetal alcohol syndrome | Q86.0 |
| Fetus and newborn affected by maternal use of alcohol | P04.3 |
| Alcohol poisoning | X45, Y15 |
| Suicide by exposure to alcohol | X65 |
| Esophageal varices | I85 |
| Gastroesophageal hemorrhage | K22.6 |
| Liver cirrhosis, unspecified | K74.0-K74.2, K74.6, K76.0, K76.7, K76.9 |
| Portal hypertension | K76.6 |
| Atrial fibrillation | I48 |
| Cancer, breast (females only) | C50 |
| Cancer, colorectal | C18, C20 |
| Cancer, esophageal | C15 |
| Cancer, laryngeal | C32 |
| Cancer, liver | C22 |
| Cancer, oral cavity and pharynx | C01-C06, C09-C10, C12-C14 |
| Cancer, pancreatic | C25 |
| Cancer, prostate (males only) | C61 |
| Cancer, stomach | C16 |
| Chronic hepatitis | K73 |
| Coronary heart disease | I20-I25 |
| Gallbladder disease | K80, K81, K83 |
| Hypertension | I10-I13, I15 |
| Infant death, low birth weight | P05.0, P07.0, P07.1 |
| Infant death, preterm birth | P07.2, P07.3 |
| Infant death, small for gestational age | P05.1 |
| Pancreatitis, acute | K85.0, K85.1, K85.3, K85.8, K85.9 |
| Pancreatitis, chronic | K86.1 |
| Pneumonia | J12-J16, J18 |
| Stroke, ischemic | G45, I63, I65-I67, I69.3 |
| Stroke, hemorrhagic | I60-I62, I69.0-I69.2 |
| Unprovoked seizures, epilepsy, or seizure disorder | G40, G41, R56.8 |
| Air-space transport | V95-V97 |
| Aspiration | W78-W79 |
| Child maltreatment | X85-X99, Y00-Y09, Y87.1 |
| Drowning injuries | W65-W70, W73, W74, Y21 |
| Fall injuries | W00-W19, Y30 |
| Fire injuries | X00-X06, X08, X09, Y26 |
| Firearm injuries | W32-W34, Y22-Y24 |
| Homicide | X85-X99, Y00-Y09, Y87.1 |
| Hypothermia | X31 |
| Motor-vehicle nontraffic crashes | V02.0, V03.0, V04.0, V09.0, V12-V14(0.0-0.2), V19.0-V19.2, V20-V28(0.0-0.2), V29.0-V29.3, V30-V39(0.0-0.3), V40-V49(0.0-0.3), V50-V59(0.0-0.3), V60-V69(0.0-0.3), V70-V79(0.0-0.3), V81.0, V82.0, V83-V86(0.4-0.7, 0.9), V88.0-V88.8, V89.0 |
| Motor-vehicle traffic crashes | V02(0.1, 0.9), V03(0.1, 0.9), V04(0.1, 0.9), V09.2, V12-V14(0.3-0.5, 0.9), V19.4-V19.6, V20-V28(0.3-0.5, 0.9), V29.4-V29.6, V29.8, V29.9, V30-V38(0.4-0.7, 0.9), V39 (0.4-0.6, 0.8, 0.9), V40-V48(0.4-0.7, 0.9), V49 (0.4-0.6, 0.8, 0.9), V50-V58(0.4-0.7, 0.9), V59 (0.4-0.6, 0.8, 0.9), V60-V68(0.4-0.7, 0.9), V69 (0.4-0.6, 0.8, 0.9), V70-V78(0.4-0.7, 0.9), V79 (0.4-0.6, 0.8, 0.9), V80.3-V80.5, V81.1, V82.1, V83-V86(0.0-0.3), V87.0-V87.8, V89.2 |
| Occupational and machine injuries | W24-W31, W45 |
| Other road vehicle crashes | V01, V05-V06, V09.1, V09.3, V09.9, V10-V11, V15-V18, V19.3, V19.8-V19.9, V80.0-V80.2, V80.6-V80.9, V81.2-V81.9, V82.2-V82.9, V87.9, V88.9, V89.1, V89.3, V89.9 |
| Poisoning (not alcohol) | X40-X44, X46-X49, Y10-Y14, Y16-Y19 |
| Suicide | X60-X64, X66-X84, Y87.0 |
| Water transport | V90-V94 |

**Appendix A2.** International Classification of Diseases (ICD-10) codes for acute or chronic conditions which are wholly or partially attributable to alcohol with an alcohol attributable fraction (AAF) ≥ 15%.

| **Cause of death** | **ICD-10 Code** | **AAF** |
| --- | --- | --- |
| Alcohol-induced pseudo-Cushing’s syndrome | E24.4 | 1.00 |
| Alcoholic psychoses | F10.0, F10.3–F10.9 | 1.00 |
| Alcohol abuse | F10.1 | 1.00 |
| Alcoholic cardiomyopathy | I42.6 | 1.00 |
| Alcoholic gastritis | K29.2 | 1.00 |
| Alcohol-induced pancreatitis | K85.0–K85.1, K85.8–K85.9 | 1.00 |
| Accidental poisoning by alcohol | T51, X45, Y15 | 1.00 |
| Intentional self-poisoning by alcohol | T51, X65 | 1.00 |
| Liver cirrhosis | K70,K74 | 0.48 |
| Oral cavity and pharynx cancer | C15, D00.1 | 0.25 |
| Tuberculosis | A15 – A19 | 0.20 |
| Liver cancer | C22, D01.5 | 0.17 |
| Falls | W00-W19, Y30 | 0.15 |
| Drowning | W65–W74 | 0.15 |
| Fires | X00–X09, Y26 | 0.15 |
| Accidental poisoning by substances other than alcohol | T36–T50, T52–T65, T96–T97, X40–X44, X46–X49, Y10–Y14, Y16–Y19 | 0.15 |
| Other unintentional injuries | V2*,W20–W64, W75–W84, X10–X33, Y20, Y22-Y25, Y27–Y29, Y31–Y34, Y85.9, Y86, Y87.2, Y89.9 | 0.15 |
| Intentional self-poisoning by substances other than alcohol | T36–T50,T52–T65, T96–T97, X60–X64, X66–X69 | 0.15 |
| Other intentional self-harm | X70-X84, Y87.0 | 0.15 |
| Assault / homicide | X85–Y09, Y87.1 | 0.15 |
| Other intentional injuries | Y35, Y89.0 | 0.15 |
| Laryngeal cancer | C32, D02.0 | 0.15 |

**Appendix B1.** Summary of the Cox Proportional Hazard Models estimating the association between alcohol consumption and mortality.

|  | **Men** | | | | |  | **Women** | | | | |  | **Combined** | | | | |
| --- | --- | --- | --- | --- | --- | --- | --- | --- | --- | --- | --- | --- | --- | --- | --- | --- | --- |
|  |  |  |  | **95% CI** | |  |  |  |  | **95% CI** | |  |  |  |  | **95% CI** | |
| **Model** | **HR** | **SE** | ***p*** | **LL** | **UL** |  | **HR** | **SE** | ***p*** | **LL** | **UL** |  | **HR** | **SE** | ***p*** | **LL** | **UL** |
| **Outcome: All-cause mortality** | | | | | | | | | | | | | | | | | |
| Self-report |  |  |  |  |  |  |  |  |  |  |  |  |  |  |  |  |  |
| Model 1 | 1.014 | 0.001 | < 0.001 | 1.012 | 1.017 |  | 1.027 | 0.005 | < 0.001 | 1.018 | 1.037 |  | 1.016 | 0.001 | < 0.001 | 1.013 | 1.018 |
| Model 2 | 1.013 | 0.001 | < 0.001 | 1.011 | 1.016 |  | 1.031 | 0.004 | < 0.001 | 1.022 | 1.040 |  | 1.015 | 0.001 | < 0.001 | 1.012 | 1.018 |
| Model 3 | 1.012 | 0.001 | < 0.001 | 1.010 | 1.015 |  | 1.032 | 0.004 | < 0.001 | 1.023 | 1.040 |  | 1.014 | 0.001 | < 0.001 | 1.012 | 1.017 |
| Model 4 | 1.008 | 0.001 | < 0.001 | 1.005 | 1.010 |  | 1.020 | 0.004 | < 0.001 | 1.011 | 1.029 |  | 1.009 | 0.001 | < 0.001 | 1.007 | 1.012 |
| Sales-corrected ^a^ |  |  |  |  |  |  |  |  |  |  |  |  |  |  |  |  |  |
| Model 1 | 1.007 | 0.001 | < 0.001 | 1.006 | 1.009 |  | 1.014 | 0.002 | < 0.001 | 1.009 | 1.019 |  | 1.008 | 0.001 | < 0.001 | 1.007 | 1.009 |
| Model 2 | 1.007 | 0.001 | < 0.001 | 1.005 | 1.008 |  | 1.015 | 0.002 | < 0.001 | 1.011 | 1.020 |  | 1.008 | 0.001 | < 0.001 | 1.006 | 1.009 |
| Model 3 | 1.006 | 0.001 | < 0.001 | 1.005 | 1.008 |  | 1.016 | 0.002 | < 0.001 | 1.012 | 1.020 |  | 1.007 | 0.001 | < 0.001 | 1.006 | 1.009 |
| Model 4 | 1.004 | 0.001 | < 0.001 | 1.003 | 1.005 |  | 1.010 | 0.002 | < 0.001 | 1.006 | 1.015 |  | 1.005 | 0.001 | < 0.001 | 1.003 | 1.006 |
| **Outcome: Alcohol-related mortality** | | |  |  |  |  |  |  |  |  |  |  |  |  |  |  |  |
| Self-report |  |  |  |  |  |  |  |  |  |  |  |  |  |  |  |  |  |
| Model 1 | 1.011 | 0.002 | < 0.001 | 1.007 | 1.015 |  | 1.018 | 0.006 | 0.003 | 1.006 | 1.030 |  | 1.012 | 0.002 | < 0.001 | 1.008 | 1.016 |
| Model 2 | 1.010 | 0.002 | < 0.001 | 1.007 | 1.014 |  | 1.022 | 0.005 | < 0.001 | 1.011 | 1.033 |  | 1.012 | 0.002 | < 0.001 | 1.008 | 1.015 |
| Model 3 | 1.010 | 0.002 | < 0.001 | 1.006 | 1.013 |  | 1.023 | 0.005 | < 0.001 | 1.013 | 1.034 |  | 1.011 | 0.002 | < 0.001 | 1.008 | 1.014 |
| Model 4 | 1.005 | 0.002 | 0.010 | 1.001 | 1.009 |  | 1.013 | 0.006 | 0.016 | 1.002 | 1.025 |  | 1.006 | 0.002 | 0.001 | 1.003 | 1.010 |
| Sales-corrected ^a^ |  |  |  |  |  |  |  |  |  |  |  |  |  |  |  |  |  |
| Model 1 | 1.006 | 0.001 | < 0.001 | 1.004 | 1.008 |  | 1.009 | 0.003 | 0.003 | 1.003 | 1.015 |  | 1.006 | 0.001 | < 0.001 | 1.004 | 1.008 |
| Model 2 | 1.005 | 0.001 | < 0.001 | 1.003 | 1.007 |  | 1.011 | 0.003 | < 0.001 | 1.006 | 1.016 |  | 1.006 | 0.001 | < 0.001 | 1.004 | 1.008 |
| Model 3 | 1.005 | 0.001 | < 0.001 | 1.003 | 1.007 |  | 1.012 | 0.003 | < 0.001 | 1.007 | 1.017 |  | 1.006 | 0.001 | < 0.001 | 1.004 | 1.007 |
| Model 4 | 1.003 | 0.001 | 0.010 | 1.001 | 1.005 |  | 1.007 | 0.003 | 0.016 | 1.001 | 1.012 |  | 1.003 | 0.001 | 0.001 | 1.001 | 1.005 |
| **Outcome: Mortality due to a condition where AAF ≥15% ^b^** | | | | | |  |  |  |  |  |  |  |  |  |  |  |  |
| Self-report |  |  |  |  |  |  |  |  |  |  |  |  |  |  |  |  |  |
| Model 1 | 1.023 | 0.002 | < 0.001 | 1.018 | 1.027 |  | 1.053 | 0.008 | 0.000 | 1.037 | 1.069 |  | 1.024 | 0.002 | < 0.001 | 1.019 | 1.028 |
| Model 2 | 1.021 | 0.002 | < 0.001 | 1.016 | 1.025 |  | 1.050 | 0.007 | 0.000 | 1.036 | 1.064 |  | 1.022 | 0.002 | < 0.001 | 1.017 | 1.026 |
| Model 3 | 1.019 | 0.003 | < 0.001 | 1.014 | 1.024 |  | 1.050 | 0.008 | 0.000 | 1.035 | 1.065 |  | 1.020 | 0.003 | < 0.001 | 1.015 | 1.025 |
| Model 4 | 1.016 | 0.003 | < 0.001 | 1.011 | 1.021 |  | 1.041 | 0.009 | 0.000 | 1.024 | 1.058 |  | 1.017 | 0.003 | < 0.001 | 1.012 | 1.022 |
| Sales-corrected ^a^ |  |  |  |  |  |  |  |  |  |  |  |  |  |  |  |  |  |
| Model 1 | 1.011 | 0.001 | < 0.001 | 1.009 | 1.014 |  | 1.025 | 0.004 | 0.000 | 1.018 | 1.032 |  | 1.012 | 0.001 | < 0.001 | 1.010 | 1.014 |
| Model 2 | 1.010 | 0.001 | < 0.001 | 1.008 | 1.013 |  | 1.025 | 0.004 | 0.000 | 1.018 | 1.032 |  | 1.011 | 0.001 | < 0.001 | 1.009 | 1.013 |
| Model 3 | 1.010 | 0.001 | < 0.001 | 1.007 | 1.012 |  | 1.025 | 0.004 | 0.000 | 1.017 | 1.032 |  | 1.010 | 0.001 | < 0.001 | 1.008 | 1.013 |
| Model 4 | 1.008 | 0.001 | < 0.001 | 1.006 | 1.011 |  | 1.021 | 0.004 | 0.000 | 1.012 | 1.029 |  | 1.009 | 0.001 | < 0.001 | 1.006 | 1.011 |

*Note.* CI, confidence interval; HR, hazard ratio. LL and UL indicate the lower and upper limits of a confidence interval, respectively. AAF = Alcohol-attributable fraction, which denotes the proportion of a health outcome which is caused by alcohol. In continuous models, the HR reflects the change in mortality risk per unit increase in consumption.

Model 1 = Includes CCHS cycle, province, rurality, age, sex and race as covariates.

Model 2 = Model 1 + self-perceived health.

Model 3 = Model 2 + education and income adequacy.

Model 4 = Model 3 + smoking status.

^a^ Data were adjusted for underreporting of alcohol use using alcohol sales data.
